# Generation and CRISPR/Cas9 editing of transformed progenitor B cells as a pseudo-physiological system to study DNA repair gene function in V(D)J recombination

**DOI:** 10.1016/j.jim.2017.08.007

**Published:** 2017-12

**Authors:** Hélène Lenden Hasse, Chloé Lescale, Joy J. Bianchi, Wei Yu, Marie Bedora-Faure, Ludovic Deriano

**Affiliations:** a Genome Integrity, Immunity and Cancer Unit, Department of Immunology, Department of Genomes and Genetics, Institut Pasteur, 75015 Paris, France; b Cellule Pasteur, University of Paris René Descartes, Sorbonne Paris Cité, Paris 75015, France

**Keywords:** V(D)J recombination, Recombination-activating gene (RAG) endonuclease, Nonhomologous end joining (NHEJ), *v*-*Abl* transformed pro-B cells, CRISPR/Cas9-mediated gene knock-out

## Abstract

Antigen receptor gene assembly is accomplished in developing lymphocytes by the V(D)J recombination reaction, which can be separated into two steps: DNA cleavage by the recombination-activating gene (RAG) nuclease and joining of DNA double strand breaks (DSBs) by components of the nonhomologous end joining (NHEJ) pathway. Deficiencies for NHEJ factors can result in immunodeficiency and a propensity to accumulate genomic instability, thus highlighting the importance of identifying all players in this process and deciphering their functions. *Bcl2* transgenic *v*-*Abl* kinase-transformed pro-B cells provide a pseudo-physiological cellular system to study V(D)J recombination. Treatment of *v*-*Abl*/*Bcl2* pro-B cells with the Abl kinase inhibitor Imatinib leads to G1 cell cycle arrest, the rapid induction of *Rag1*/*2* gene expression and V(D)J recombination. In this system, the *Bcl2* transgene alleviates Imatinib-induced apoptosis enabling the analysis of induced V(D)J recombination. Although powerful, the use of mouse models carrying the *Bcl2* transgene for the generation of *v*-*Abl* pro-B cell lines is time and money consuming. Here, we describe a method for generating *v*-*Abl*/*Bcl2* pro-B cell lines from wild type mice and for performing gene knock-out using episomal CRISPR/Cas9 targeting vectors. Using this approach, we generated distinct NHEJ-deficient pro-B cell lines and quantified V(D)J recombination levels in these cells. Furthermore, this methodology can be adapted to generate pro-B cell lines deficient for any gene suspected to play a role in V(D)J recombination, and more generally DSB repair.

## 1 Introduction

Mammalian cells employ two canonical mechanisms to repair DNA double-strand breaks: homologous recombination (HR) and nonhomologous end joining (NHEJ) (Symington and Gautier, 2011). HR requires a template – the chromatid sister or homolog – to direct repair and is active during the S/G2 cell cycle phase. In contrast, NHEJ directly ligates DSBs with short (typically 1–4 nucleotides) or no homologies. NHEJ appears to be the dominant DSB repair pathway used in mammalian cells and is active throughout the cell cycle, particularly in G0/G1. During NHEJ (Deriano and Roth, 2013), the Ku70/80 heterodimer (Ku) specifically recognizes DSB ends and recruits the DNA-dependent protein kinase catalytic subunit (DNA-PKcs) to form the DNA-PK holoenzyme. DNA-PK phosphorylates multiple substrates, promoting synapsis of DNA ends and facilitating the recruitment of end processing enzymes such as the Artemis endonuclease. Finally, DNA ligase IV in complex with XRCC4 and XRCC4-like factor (XLF, also called Cernunnos or NHEJ1), a protein structurally related to XRCC4, performs ligation of DNA ends. PAXX, PAralog of XRCC4 and XLF, is a third XRCC4-like protein and is the most recently identified NHEJ factor (Craxton et al., 2015, Ochi et al., 2015, Xing et al., 2015). PAXX promotes DSB repair via its interaction with Ku and shares a function with XLF that is critical for DSB joining (Balmus et al., 2016, Kumar et al., 2016, Lescale et al., 2016b, Tadi et al., 2016, Hung et al., 2017, Liu et al., 2017). Based on their requirement for DSB joining in all settings and their evolutionary conservation, Ku, XRCC4 and Ligase IV are considered core NHEJ factors.

NHEJ is essential for V(D)J recombination as illustrated by the severe combined immunodeficiency observed in some human patients and mouse models with NHEJ defects (de Villartay, 2009). V(D)J recombination takes place in G1-arrested progenitor B and T lymphocytes and is initiated by the lymphoid-specific RAG1/2 endonuclease, which recognizes specific recombination signal sequences (RSSs) flanking V, D, and J coding segments (Schatz and Swanson, 2011). Cleavage by RAG generates two different end structures: 5′ phosphorylated blunt signal ends and covalently closed hairpin coding ends. These ends are then joined by NHEJ in a recombinant configuration, forming a coding joint (the rearranged antigen receptor gene) and a reciprocal product termed a signal joint. The core factors, Ku, XRCC4, and Ligase 4 are required for both coding and signal joint formation while DNA-PKcs/Artemis are necessary for coding end processing prior to ligation (Rooney et al., 2004, Helmink and Sleckman, 2012, Deriano and Roth, 2013). While XLF is required for repair of DSBs induced by genotoxic stress, it is dispensable for the repair of RAG-generated DSBs in lymphoid cells due to overlapping activities with additional factors or complexes. One such complex is the ataxia telangiectasia mutated (ATM) kinase-dependent DNA damage response. Specifically, while not essential for V(D)J recombination, loss of ATM (or its substrates H2AX or 53BP1) leads to a block in repair of RAG-DSBs in XLF-deficient lymphoid cells (Zha et al., 2011, Kumar et al., 2014). Similarly, PAXX/XLF double deficiency abolishes the repair of RAG-DSBs even though the singular loss of these paralogs does not lead to major NHEJ defects in lymphoid cells (Kumar et al., 2016, Lescale et al., 2016b, Hung et al., 2017, Liu et al., 2017). Interestingly, expression of a mutant form of RAG2, lacking the C-terminal regulatory portion of the protein, in XLF-deficient lymphocytes leads to a dramatic defect in V(D)J recombination due to a block in NHEJ, indicating that the RAG recombinase participates in repair of RAG-generated DNA breaks in recombining lymphocytes (Lescale et al., 2016a, Lescale and Deriano, 2017).

Altogether, as highlighted by the data outlined above, the V(D)J recombination reaction is cell cycle stage specific (i.e. G1-arrested progenitor lymphocytes), generates unique structures of DNA ends (i.e. coding and signal ends) and relies on NHEJ, and, to a lesser extent, the ATM-dependent DNA damage response machinery. It thus provides a unique physiological system to discover novel DSB repair factors and elucidate their precise functions. Accordingly, *v*-*Abl* pro-B cell lines that enable the manipulation and study of V(D)J recombination ex vivo are a powerful tool for gaining a fuller understanding of DSB repair.

Expression of the protein tyrosine kinase encoded by the viral (v)-*Abl* oncogene of Abelson murine leukemia virus induces transformation of early B cells (Rosenberg et al., 1975). Fundamental insights into the mechanisms which regulate the early steps of B cell development, specifically the sequential rearrangement of immunoglobulin genes, have emerged from analysis of these *v*-*Abl* transformed B cell lines (Alt et al., 1986). *v*-*Abl* progenitor B cell transformants (*v*-*Abl* pro-B cells) typically display cell surface markers characteristic of a pro- or early pre-B stage of development (B220^+^ CD43^+^ CD25^−^ IgM^−^). They are also characterized by a rapid proliferative state, low expression of *Rag1* and *Rag2* and infrequent immunoglobulin light chain gene rearrangement. Treatment of *v*-*Abl* pro-B cell lines with the Abl kinase inhibitor Imatinib (also named STI-571 or Glivec) leads to G1 arrest, rapid induction of *Rag1* and *Rag2* expression and RAG-mediated DNA breakage at immunoglobulin light chain genes (Muljo and Schlissel, 2003). STI-571 treatment also triggers apoptosis in these cells within 24 to 36 h thus limiting their usage for complete analysis of V(D)J recombination intermediates and products (Muljo and Schlissel, 2003). This limitation is overcome in *v*-*Abl* pro-B cell lines generated from mice expressing an Eμ-*Bcl2* transgene (Bredemeyer et al., 2006). In this setting, the *Bcl2* transgene circumvents STI-571-induced apoptosis thus G1-arrested *v*-*Abl* pro-B cells can be maintained in culture for up to five days without significant cell death enabling the analysis of induced V(D)J recombination at endogenous RAG target loci as well as any chromosomally integrated reporter substrate (Bredemeyer et al., 2006). Although powerful, the use of mouse models carrying the *Bcl2* transgene for the generation of *v*-*Abl* pro-B cell lines is time and money consuming.

Here, we describe a method for generating *v*-*Abl*/*Bcl2* pro-B cell lines from wild type mice and for performing gene knock-out using episomal CRISPR (clustered regularly interspaced short palindromic repeats)/Cas9 targeting vectors. In this system, the anti-apoptotic *Bcl2* gene is retrovirally introduced into *v*-*Abl* transformed pro-B cells thus bypassing the need of *Bcl2* transgenic animals. Additionally, CRISPR/Cas9-mediated gene knock-out can be achieved in approximately 4 weeks, facilitating the interrogation of candidate V(D)J recombination genes. Using this approach, we generated distinct NHEJ-deficient pro-B cell lines and quantified V(D)J recombination levels in these cells (Lescale et al., 2016b). Furthermore, this methodology can be adapted to generate pro-B cell lines deficient for any gene suspected to play a role in V(D)J recombination, and more generally DSB repair.

## 2 Materials and methods

### 2.1 Mice

3- to 6-week-old C57BL/6J mice (JAX Stock Number 000664) were used for generating wild type *v*-*Abl*/*Bcl2* pro-B cell lines.

### 2.2 Preparation of retroviral supernatants

Retroviral supernatants were prepared by transfection of Platinum-E cells (Morita et al., 2000) with either pMSCV-*v*-*Abl* plasmid encoding for Abl (Bredemeyer et al., 2006), pMSCV-*Bcl2*-IRES-*puro* plasmid encoding for Bcl2 and puromycin selectable marker (Kollmann et al., 2011) or pMX-RSS-GFP/IRES-hCD4 (pMX-INV) reporter plasmid encoding for the cell surface marker human CD4 (Liang et al., 2002, Bredemeyer et al., 2006) using Lipofectamine 2000 (Invitrogen), harvested 48 h and 72 h after transfection, snap frozen and conserved at − 80 °C.

### 2.3 Establishment of a wild type *v*-*Abl*/*Bcl2* transformed pro-B cell line

**Fig. 1:**
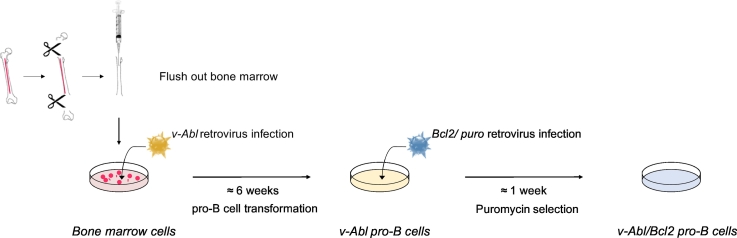
Generation of *v*-*Abl*/*Bcl2* transformed pro-B cell lines. Total bone marrow from 3- to 6-week-old mice are flushed and infected with a retrovirus encoding for the *v*-*Abl* oncogene. Cells are cultured for 6 weeks until homogeneous *v*-*Abl* transformed pro-B cells are obtained. *v*-*Abl* pro-B cells are then transduced with a retrovirus encoding the *Bcl2* anti-apoptotic gene and the *Puromycin* resistance gene. *v*-*Abl*/*Bcl2* pro-B cells are selected by adding Puromycin to the medium for one week (see text for details).

One mouse femur was isolated, washed five times in Dulbeccos's phosphate-buffered saline (PBS) (Gibco) with penicillin (100 U/ml)/streptomycin (100 μg/ml) (Gibco) and cut at both ends near the joints (Fig. 1). The bone marrow was flushed out from both ends with a 25-gauge needle in a 15 ml Falcon containing 10 ml of RPMI 1640 supplemented with Glutamine (Gibco), 15% fetal bovine serum (FBS) (Sigma, #F6178), penicillin (100 U/ml)/streptomycin (100 μg/ml) and 50 μM 2-mercaptoethanol (Gibco). The suspension was mixed gently several times using a 5 ml pipette and bone pieces and debris were left to settle to the bottom of Falcon tube for 2 min. Approximately 9 ml of the top suspension were removed and spun at 1200 rpm 5 min (Eppendorf Centrifuge 5810 R, Rotor A-4-81) at room temperature in a new 15 ml tube. Liquid was gently removed and the cell pellet was resuspended in 1,5 ml of media and distributed into 3 wells of a 6-well plate as follows - well 1: no dilution (1 ml cell suspension), well 2: 2.4-fold dilution (0.5 ml cell suspension + 0.7 ml media) and well 3: 12-fold dilution (0.2 ml of cell suspension from well 2 + 0.8 ml of media). 1 ml of *v*-*Abl* retroviral supernatant (see Section 2.2) was then added to each well and cells were spinoculated at 2000 rpm at 30 °C for 1 h (Eppendorf Centrifuge 5810 R, Rotor A-4-81) in the presence of 5 μg/ml polybrene (Sigma, #TR-1003 EMD Millipore). 2 ml of fresh media were added to each well one day after spinoculation of bone marrow cells. 7 to 10 days after infection, clusters of transformed cells grew at the bottom of the well and were fed with 2 ml of fresh media. Cells were allowed to grow until fairly dense and split 1:2 into adjacent well until having two confluent wells. Cells were subsequently split 1:2 to 1:3 into small T flasks for an additional three to four weeks. *v*-*Abl* transformed pro-B cell aliquots were frozen in freezing media containing 90% FBS/10% dimethylsulfoxide (DMSO, Sigma) twice during the transformation period. 1 ml of culture media containing 2 × 10^6^ *v*-*Abl* transformed pro-B cells were subsequently transduced with 1 ml of *Bcl2*/*puro* retroviral supernatant (see Section 2.2) (Fig. 1). Two days after infection, transduced cells were selected by adding puromycin at 3 μg/ml (Sigma) to the media and cultured for approximately one week. *v*-*Abl*/*Bcl2* (hereafter named *v*-*Abl*) pro-B cells were further cultured in RPMI 1640 supplemented with Glutamine, 10% FBS, penicillin (100 U/ml)/streptomycin (100 μg/ml) and 50 μM 2-mercaptoethanol and frozen in 90% FBS/10% DMSO.

### 2.4 Generation of NHEJ-deficient *v*-*Abl* pro-B cells by CRISPR/Cas9 gene editing

#### 2.4.1 CRISPR/Cas9 expression plasmids

**Fig. 2:**
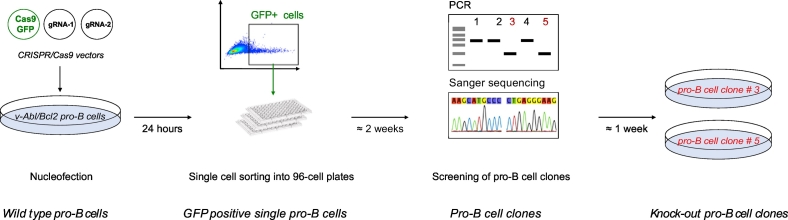
Generation of knock-out pro-B cell clones. 15 million pro-B cells are transfected with plasmids encoding for Cas9-GFP and specific gRNAs. After 24 h in culture media, nucleofected cells (GFP^+^ cells) are single-cell sorted into 96-well plates. Approximately two weeks later, isolated clones are screened for the presence of inactivating mutations using PCR amplification and sanger sequencing. Pro-B cell clones containing homozygous inactivating mutations are further cultured and functionally assayed (see text for details).

Cas9 was expressed from plasmid pCas9-GFP (Addgene plasmid #44719) and guide RNAs (gRNAs) were expressed from plasmid MLM3636 (Addgene plasmid # 43860) (Fig. 2). Two gRNAs for each gene were used to delete one or multiple exons containing the ATG starting codon or encoding for a specific structural/catalytic domain (Table 1). The choice of gRNA targets was based on high target specificity and low number of off-target sites, as determined using the online CRISPR Design tool (http://crispr.mit.edu/). Two gRNA-related oligonucleotides (A and B) containing the 18 to 20 bp gRNA sequence and the linker sequences (5′-ACACCG-gRNA-G-3′ for the oligonucleotide A and 5′-AAAAC-gRNA′-CG-3′ for the oligonucleotide B) were used for cloning into the *Bsm*BI cloning site of MLM3636. Briefly, annealing of gRNA-related oligonucleotides A and B (100 μM stock) was performed by mixing 10 μl of each oligonucleotide (Final oligo concentration: 10 μM) with 80 μl of buffer containing 10 mM Tris pH 7,5, 1 mM EDTA and 50 mM NaCl and incubating at 95 °C for 15 min and then at room temperature for at least 1 h. MLM3636 plasmid was digested by *Bsm*BI enzyme (NEB). Linearized MLM3636 plasmid was run on a 1% gel, extracted and purified (Qiagen, #28704). Ligation was performed by incubating 100 ng of linearized MLM3636 plasmid with 0.05 μM annealed gRNA-related oligonucleotides, 2 μl 10 × Ligase buffer (NEB) and 1 μl T4 Ligase (NEB) in a 20 μl volume reaction for 1 h at room temperature. DH5α bacteria (Invitrogen) were transformed with the ligation product and amplified. Purified plasmids were Sanger sequenced using LKO.1 5′ primer (5′-GACTATCATATGCTTACCGT-3′) to verify correct gRNA cloning.

**Table 1:** CRISPR oligonucleotide and PCR primer sequences. *Linker sequences for cloning into *Bsm*BI-disgested MLM3636 are underlined; **gRNA score and predicted off-targets are determined using the online CRISPR Design tool http://crispr.mit.edu/ website from the Zhang lab.

Oligonucleotide name	Oligonucleotide sequence (5′-> 3′)*	sgRNA target coordinates (*NCBI37*/*mm10*)	Score**	Offtargets (in genes), score ≥ 1**	Deletion	Primer name	Primer sequence (5′-> 3′)	Expected PCR size wild type allele	Expected PCR size CRISPR/Cas9 edited allele
Paxx gRNA-1 A	ACACCGACTAGAGGTTGAAGTCGTCGG	chr2: 25,460,785–25,460,804	94	0 (0)	Part of exon 1–4 ≥ conformation changes and frameshift and/or stop mutations	Paxx PCR primer 1	ATGAGAGACTCCCCTGGACA	1299 bp	673 bp
Paxx gRNA-1 B	AAAACCGACGACTTCAACCTCTAGTCG								
Paxx gRNA-2 A	ACACCGATGCAACCTAGAGAGGCGGCG	chr2: 25,460,173–25,460,192	78	3 (1)		Paxx PCR primer 2	ACCCGGAAACAATGTCAACC		
Paxx gRNA-2 B	AAAACGCCGCCTCTCTAGGTTGCATCG								
Xrcc4 gRNA-1 A	ACACCGGAATGTATAACAGGAGACGGG	chr13: 90,062,346–90,062,365	79	1 (0)	Part of the XRCC4 functional core region	Xrcc4 PCR primer 1	GGCTGACAGTCTGAGGCTAT	1078 bp	790 bp
Xrcc4 gRNA-1 B	AAAACCCGTCTCCTGTTATACATTCCG								
Xrcc4 gRNA-2 A	ACACCGGCCGAGACTCCTTAGAAAAGG	chr13: 90,062,044–90,062,063	65	10 (1)		Xrcc4 PCR primer 2	GCCTCCATGTCAGTACTGGT		
Xrcc4 gRNA-2 B	AAAACCTTTTCTAAGGAGTCTCGGCCG								
Ligase 4 gRNA-1 A	ACACCGCACTGGGGGTTCGGTAATTGG	chr8: 9,973,430–9,973,449	89	1 (0)	Active-site lysine and conserved catalytic domains found in all ATP dependent DNA ligases	Ligase 4 PCR primer 1	ACAGTATGTATCCGGGCCTG	2062 bp	557 bp
Ligase 4 gRNA-1 B	AAAACCAATTACCGAACCCCCAGTGCG								
Ligase 4 gRNA-2 A	ACACCGACCTACATGAAGGTGTTTCGG	chr8: 9,971,925–9,971,944	81	1 (0)		Ligase 4 PCR primer 2	TCATGTCCCCTTTGCAGACT		
Ligase 4 gRNA-2 B	AAAACCGAAACACCTTCATGTAGGTCG								
XLF gRNA-1 A	ACACCGTTAGCATACACCAACTTCG	chr1: 75,046,363–75,046,380	78	2 (0)	Whole exon 1	Xlf PCR primer 1	ACAAGGTCTAATGCACCCCA	753 bp	436 bp
XLF gRNA-1 B	AAAACGAAGTTGGTGTATGCTAACG								
XLF gRNA-2 A	ACACCGCACCAACAGGTACTCATAG	chr1: 75,046,668–75,046,685	84	1 (0)		Xlf PCR primer 2	GGGTTGCAGCCTTAGAAAAGT		
XLF gRNA-2 B	AAAACTATGAGTACCTGTTGGTGCG								

#### 2.4.2 CRISPR/Cas9 editing of wild type *v*-*Abl*/*Bcl2* pro-B cells

##### 2.4.2.1 Nucleofection and cell sorting

15 million pro-B cells were nucleofected using the Cell Line Nucleofector® Kit V from Lonza (program X-001, Amaxa Nucleofector Technology) and 6 μg of plasmid mix (2 μg of each plasmid; two pMLM3636-gRNA plasmids and the pCas9-GFP plasmid) (Fig. 2). Electroporated cells were quickly diluted in 37 °C pre-warmed culture media at a density of 1.5 × 10^6^ cells/ml and left to recover for 24 to 36 h. The next day, single cells expressing GFP were sorted using a BD FACSAria™ cell sorter into round bottom 96-well plates containing 200 μl of culture media. The percentage of GFP positive cells was typically low (2 to 5%) but sufficient to sort up to 384 cells into four 96-well plates (Fig. 2). Plates containing nucleofected cells were then kept in culture for up to two weeks until clones could be identified by eye. At this stage, pro-B cell clones were transferred to a 12-well plate containing 2.5 ml of culture media in each well. When cells reached confluence (approximately 1 to 2 × 10^6^ cells/ml), 1 ml was transferred into a 2 ml Eppendorf tube. The tube was spun at 10,000 rpm for 2 min (Eppendorf Centrifuge 5424, Rotor FA-45-24-11) and genomic DNA was prepared as described in Section 2.4.2.2. The remaining cells were diluted 1:5 and cultured until positive clones (those containing a homozygous inactivating mutation) were identified and selected.

##### 2.4.2.2 Genomic DNA extraction and PCR screening

The cell pellet (containing approximately 1 × 10^6^ cells) was resuspended in 1 ml of lysis buffer containing 0.2 M NaCl, 0.4% SDS, 0.1 M Tris pH 7.5, 0.01 M EDTA pH 8 and 0.4 mg/ml proteinase K (Eurobio, #GEXPRK01-B5) and incubated at 55 °C for a minimum of 1 h. The tube was then left to cool down to room temperature. 1 ml of isopropanol was added to the lysis buffer and the tube was mixed thoroughly until genomic DNA precipitate formed. The tube was centrifuged at 14,000 rpm for 2 min (Eppendorf Centrifuge 5424, Rotor FA-45-24-11) and the supernatant was removed. The DNA pellet was washed once with 70% Ethanol (14,000 rpm centrifugation for 2 min) and air dried for approximately 15 min. Genomic DNA preparation was suspended in 400 μl 0.1 × TE solution. For PCR screening, 5 μl of genomic DNA was mixed with 5 μl 10 × Taq buffer (Invitrogen, #18038042), 1 μl 50 mM MgCl2 (Invitrogen, #18038042), 5 μl 750 μM dNTP, 1 μl 10 μM primer 1, 1 μl 10 μM primer 2, 0.5 μl Taq polymerase (5 U/μl, Invitrogen, #18038042) and 30 μl H_2_O (see Table 1 for primer list and sequences). The following PCR reactions was run: 1 ×(95 °C-30 s) 30 ×(95 °C-30 s, 60 °C-40 s, 72 °C-30 s) 1 ×(72 °C-5 min) 1 ×(4 °C-forever). Annealing temperature was adapted depending on the Tm of each primer 1/2 couple. Elongation time was adapted depending on the expected amplicon size. 25 μl of PCR product was run on a 2% agarose gel and bands that differ in size to the control germline band were gel extracted and purified (Qiagen, #28704) (Fig. 2). Purified PCR products were Sanger sequenced using PCR primer 1 and PCR primer 2 to identify CRISPR/Cas9-induced deletions and insertions at the cleavage site. Clones for which inactivating mutations (leading to the deletion of the ATG or a core domain of the protein without producing a chimeric protein) were identified on both alleles were selected for functional analysis (see Section 2.5 V(D)J recombination assay). A minimum of two independent knock-out clones were assayed for V(D)J recombination and compared to the non-edited mother *v*-*Abl* pro-B cell line.

### 2.5 V(D)J recombination assay

**Fig. 3:**
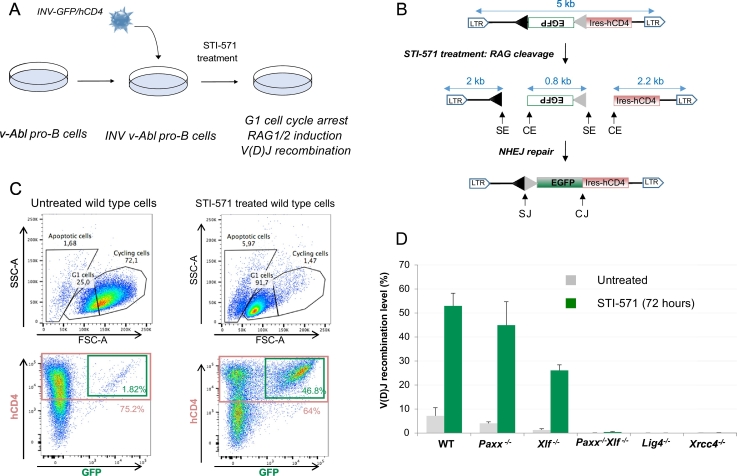
Analysis of V(D)J recombination in *v*-*Abl* pro-B cell lines. A. The pMX-INV (INV-GFP/hCD4) substrate is introduced in *v*-*Abl* pro-B cells by retroviral infection and cells that have integrated the recombination substrate are enriched based on human CD4 (hCD4) expression. To assay V(D)J recombination, cells are treated with the Abelson kinase inhibitor Imatinib (STI-571) for 72 h which leads to G1 cell cycle arrest, induction of RAG1 and RAG2 expression and V(D)J recombination. B. Schematic of the pMX-INV recombination substrate. The 12-recombination signal sequence (RSS-12; black triangle), GFP cDNA, 23-recombination signal sequence (RSS-23; grey triangle), IRES - human CD4 cDNA (IRES-hCD4), long terminal repeat sequence (LTR), coding end (CE), signal end (SE), coding joint (CJ) and signal joint (SJ) are shown. The sizes of the recombination cassette and the recombination intermediates are indicated. C. Upper panel. SSC-A/FSC-A gating of untreated and STI-571 treated (72 h) wild type *v*-*Abl* pro-B cells. Apoptotic, G1 and cycling cells are indicated. Lower panel. V(D)J recombination efficiency is assessed by flow cytometry and scored as the percentage of GFP positive cells among hCD4 positive cells. D. V(D)J recombination efficiency in wild type and CRISPR/Cas9 knock-out *v*-*Abl* pro-B cell lines. Data represent the means ± SEMs of at least 2 independent experiments performed using 2 wild type (WT), 2 *Paxx*^−/−^, 2 *Xlf*^−/−^, 2 *Paxx*^−/−^*Xlf*^−/−^, 6 *Xrcc4*^−/−^ and 4 *Lig4*^−/−^ clones.

1 ml of culture media containing 2 × 10^6^ *v*-*Abl* pro-B cells were transduced with 1 ml of pMX-INV retroviral supernatant (Section 2.2) (Fig. 3A, B). Pro-B cells that had integrated the pMX-INV recombination substrate were enriched based on hCD4 expression using hCD4 Microbeads (Miltenyi, #130-045-101) following manufacturer instructions. Typically, 10 × 10^6^ *v*-*Abl* pro-B cells were washed once in 0.5% FBS/PBS solution and resuspended in 80 μl of the same solution. 20 μl of hCD4 Microbeads were added to the cell suspension and incubated for 20 min. Cells were then washed once in 0.5% FBS/PBS and resuspended in 1 ml of the same solution before magnetic selection using LS column (Miltenyi, #130-042-401). After a few days in culture, purified pMX-INV *v*-*Abl* pro-B cells (10^6^/ml) were treated with 3 μM of the Abl kinase inhibitor STI-571 (Novartis) or 0.3 μM of the STI-571 analogous Abl kinase inhibitor PD180970 (Sigma) for 72 h and assayed for recombination levels by FACS analysis of GFP/hCD4 expression (hCD4-PE antibody, Miltenyi, #130-091-231; 1/20 dilution) (Fig. 3C). V(D)J recombination levels were scored by FACS analysis as the percentage of GFP positive cells among total hCD4 positive cells (Fig. 3C and D).

## 3 Results

### 3.1 Generation and characterization of wild type *v*-*Abl*/*Bcl2* pro-B cell lines

We first generated *v*-*Abl*/*Bcl2* (*v*-*Abl*) pro-B cell lines from wild type C57BL/6J mice (Fig. 1) (Lescale et al., 2016a, Lescale et al., 2016b). To assess V(D)J recombination, we transduced wild type *v*-*Abl* pro-B cell lines with the pMX-RSS-GFP/IRES-hCD4 retroviral recombination substrate (pMX-INV) that enables GFP expression upon successful RAG-dependent chromosomal inversional recombination (Fig. 3A, B). Subsequent treatment of these *v*-*Abl* pro-B cell lines with the Abl kinase inhibitor Imatinib (STI-571) for 3 days led to an almost complete G1 arrest (< 2% of large sized cycling pro-B cells) without significant cell death (< 6% cell death) (Fig. 3C). In addition, flow cytometry analysis showed robust levels of pMX-INV rearrangement in wild type *v*-*Abl* pro-B cells treated with STI-571 for 3 days (53%, Fig. 3C, D) (Lescale et al., 2016a, Lescale et al., 2016b). Altogether, these results indicate that, as previously reported in the context of *v*-*Abl* pro-B cells carrying a Eμ-*Bcl2* transgene (Bredemeyer et al., 2006), deregulating expression of Bcl2 by mean of a retrovirus similarly abolishes STI-571 induced apoptosis in *v*-*Abl* pro-B cells enabling the study of V(D)J recombination in G1-arrested pro-B cells.

### 3.2 V(D)J recombination levels in CRISPR/Cas9-edited *v*-*Abl* pro-B cell clones

We next employed CRISPR/Cas9-mediated gene editing to create a number of different *v*-*Abl* pro-B cell lines deficient for NHEJ factors (Fig. 2, Table 1). We deleted *Paxx* (Δexons1–4) from wild type *v*-*Abl* pro-B cells, generating *Paxx*^−/−^ *v*-*Abl* pro-B cell clones and *Xlf* (Δexon1) from wild type and *Paxx*^−/−^ cells to generate *Xlf*^−/−^ and *Paxx*^−/−^ *Xlf*^−/−^ *v*-*Abl* pro-B cell clones, respectively (Lescale et al., 2016b). Finally, we generated pro-B cell lines deficient for the core NHEJ factors XRCC4 and Ligase 4. *Xrcc4*^−/−^ clones were generated by deleting exon3 of *Xrcc4*, which encodes part of the XRCC4 functional core region (Gao et al., 1998), from wild type *v*-*Abl* pro-B cells. *Lig4*^−/−^ clones were generated by removing nucleotide sequences that encode critical functional domains (Frank et al., 1998) from wild type *v*-*Abl* pro-B cells (Table 1, Fig. 2). We next assessed V(D)J recombination levels in these cell lines (Fig. 3). Flow cytometry analysis revealed robust levels of rearrangements in STI-571-treated wild type (WT) (53%), *Paxx*^−/−^ (45%) and *Xlf*^−/−^ (26%) cells (Fig. 3D) (Lescale et al., 2016b). Of note, we consistently found a 2-fold decrease in V(D)J recombination levels in XLF-deficient pro-B cells as compared to wild type pro-B cells, consistent with the small decrease in thymocyte and peripheral lymphocyte numbers observed in XLF-deficient mice (Li et al., 2008, Vera et al., 2012). In sharp contrast, we found severely impaired inversional rearrangement in *Paxx*^−/−^ *Xlf*^−/−^ (0.42%) cells as compared with the WT, *Paxx*^−/−^ and *Xlf*^−/−^ *v*-*Abl* pro-B cells (Lescale et al., 2016b). Notably, the intensity of the V(D)J recombination defect in *Paxx*^−/−^ *Xlf*^−/−^ cells was similar to that of *Xrcc4*^−/−^ (0.04%) and *Lig4*^−/−^ (0.02%) cells (Fig. 3D), indicating that XLF and PAXX might act during DSB joining. Thus, molecular analysis of V(D)J recombination intermediates and products in *v*-*Abl* pro-B cell lines revealed that PAXX and XLF play critical overlapping functions during NHEJ-mediated repair of DSBs in lymphocytes (Kumar et al., 2016, Lescale et al., 2016b, Hung et al., 2017, Liu et al., 2017). These studies also demonstrated the utility of CRISPR/Cas9-edited *v*-*Abl* pro-B cell lines in elucidating DSB repair gene functions.

## 4 Discussion

**Fig. 4:**
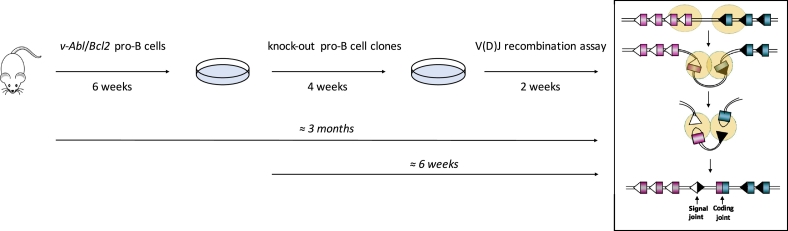
Workflow for generation of *v*-*Abl* pro-B cell lines and analysis of V(D)J recombination. Purple and blue colored boxes represent coding segments within a stereotyped immunoglobulin locus; white and black triangles represent 12- and 23-RSSs; RAG proteins are depicted as yellow circles. (For interpretation of the references to color in this figure legend, the reader is referred to the web version of this article.)

We describe here a methodology for generating *v*-*Abl* pro-B cell lines from wild type mice in approximately 6 weeks. When combined with CRISPR/Cas9 technology, *v*-*Abl* pro-B cells can be effectively used to test the role of candidate genes in V(D)J recombination (Fig. 4). Importantly, this protocol can be easily adapted with any gRNA/Cas9 expression vectors and editing strategy of choice (gene editing, gene regulation, genome-wide screening, etc.) (Sternberg and Doudna, 2015). Notably, although we focus here on the flow cytometry-based analysis of V(D)J recombination, more in-depth analysis of V(D)J recombination intermediates (i.e. analysis of broken CEs and SEs) and products (i.e. quantification and sequence analysis of CJs, SJs and hybrid joints that result from the aberrant joining of a coding end to a signal end) can be achieved by Southern blot, fluorescence in situ hybridization, and PCR amplification and sequencing using probes and primers specific to the endogenous immunoglobulin *k* locus or the chromosomally integrated pMX-INV substrate (Fig. 3B) (Bredemeyer et al., 2006, Yin et al., 2009, Helmink et al., 2011, Kumar et al., 2016, Lescale et al., 2016a, Lescale et al., 2016b, Hung et al., 2017, Liu et al., 2017).

Here, we selectively knocked-out four NHEJ genes: *Xrcc4*, *Xlf*, *Paxx* and *Ligase 4*, in *v*-*Abl* pro-B cells and assayed V(D)J recombination in these settings. V(D)J recombination reporter assays revealed severely impaired inversional rearrangement in *Xrcc4*^−/−^ and *Lig4*^−/−^ *v*-*Abl* pro-B cells (Fig. 3D), consistent with the role of the XRCC4/Ligase 4 complex in carrying out the ligation step during NHEJ (Critchlow et al., 1997, Grawunder et al., 1997). Conversely, *Xlf*^−/−^ and *Paxx*^−/−^ *v*-*Abl* pro-B cell clones sustained robust RAG-mediated inversional recombination indicating that these two factors are dispensable for repair of RAG-DSBs. Strikingly, combined loss of XLF and PAXX led to a complete block in RAG-mediated recombination, possibly due to overlapping functions between these two structurally related proteins during NHEJ (Fig. 3D) (Lescale et al., 2016b). Notably, these findings are supported by the analysis of knock-out animal models showing that PAXX and XLF single deficiency does not lead to a block in lymphocyte differentiation and *Paxx*^−/−^ and *Xlf*^−/−^ mice show relatively normal mature lymphocyte numbers in the peripheral lymphoid organs. In addition, *Paxx*^−/−^ and *Xlf*^−/−^ mice are viable, grow normally and are fertile (Li et al., 2008, Balmus et al., 2016, Liu et al., 2017). In contrast, the combined deficiency of PAXX and XLF is embryonic synthetic-lethal and associated with significant growth defects, increased genomic instability and cell death in the developing central nervous system, and a block in lymphocyte development, phenotypes that are strongly reminiscent of *Xrcc4*^−/−^ or *Lig4*^−/−^ mice (Frank et al., 1998, Gao et al., 1998, Balmus et al., 2016, Liu et al., 2017). Thus, CRISPR/Cas9 edited *v*-*Abl* pro-B cell lines provide a physiological surrogate ex vivo system to interrogate the function of candidate DNA repair factors during V(D)J recombination.

*v*-*Abl* pro-B cell lines also provide a valuable experimental system to test more general DSB response and repair activities in cycling or G1-arrested cells. For instance, over the past years, this cell system has been used to assay cell sensitivity to irradiation and genotoxic drugs, nuclear DNA damage foci formation, DNA breakage sites, translocation formation and genetic and epigenetic modifications that occur upon DSB formation and repair (Bredemeyer et al., 2006, Bredemeyer et al., 2008, Yin et al., 2009, Zhang et al., 2012, Dorsett et al., 2014, Hu et al., 2015, Canela et al., 2016, Kumar et al., 2016, Lescale et al., 2016a, Lescale et al., 2016b, Hung et al., 2017, Liu et al., 2017). With the development of CRISPR/Cas9 editing tools, it now provides a robust system with which to probe the role and mechanism of a wide repertoire of DSB response and repair factors.
